# Association between Abnormal Gait Patterns and an Elevated Degree of Pain after Daily Walking: A Preliminary Study

**DOI:** 10.3390/ijerph19052842

**Published:** 2022-03-01

**Authors:** Shogo Misu, Tsuyoshi Asai, Shunsuke Murata, Ryo Nakamura, Tsunenori Isa, Yamato Tsuboi, Kensuke Oshima, Shota Koyama, Ryuichi Sawa, Yoshihiro Fukumoto, Rei Ono

**Affiliations:** 1 Department of Physical Therapy, Faculty of Nursing and Rehabilitation, Konan Women’s University, 6-2-13, Morikita-machi, Higashinada-ku, Kobe 658-0001, Japan; 2 Department of Community Health Sciences, Kobe University Graduate School of Health Sciences, 7-10-2, Tomogaoka, Suma-ku, Kobe 654-0142, Japan; murata.s@ncvc.go.jp (S.M.); t.issssa.777@gmail.com (T.I.); yt.green1@gmail.com (Y.T.); ono@phoenix.kobe-u.ac.jp (R.O.); 3 Faculty of Rehabilitation, Kansai Medical University, 18-89 Uyamahigashicho, Hirakata 573-1136, Japan; asaits@makino.kmu.ac.jp (T.A.); fukumoty@hirakata.kmu.ac.jp (Y.F.); 4 Department of Preventive Medicine and Epidemiology, National Cerebral and Cardiovascular Center Research Institute, 6-1, Kishibe-Shimmachi, Suita 564-8565, Japan; 5 Visiting Nursing Station Sakura, 9-17, Kawanishi-cho, Nishinomiya 662-0951, Japan; nackham.pt@gmail.com; 6 Everehab, Inc., 46 Kamitakanonakamachi, Sakyo-ku, Kyoto 606-0044, Japan; ptkensuke@yahoo.co.jp; 7 Department of Rehabilitation, Saiseikai Hyogoken Hospital, 5-1-1, Fujiwaradainakamachi, Kita-ku, Kobe 651-1302, Japan; shota11.koyama@gmail.com; 8 Department of Physical Therapy, Faculty of Health Science, Juntendo University, 2-1-1, Hongo, Bunkyo-ku, Tokyo 113-0033, Japan; r.sawa.ia@juntendo.ac.jp

**Keywords:** gait, pain after walking, inertial sensor, acceleration, older adults

## Abstract

This study aimed to investigate whether abnormal gait patterns are associated with experiencing an elevated degree of pain after daily walking. In this preliminary, cross-sectional study, 223 community-dwelling older adults were assessed for pain experienced after daily walking using a simple question that involved asking the subject about their past experiences of an elevated degree of pain after walking for 400 m or more. Gait patterns were assessed using the Comprehensive Gait Assessment using InerTial Sensor score (C-GAITS score), derived from the data measured by Inertial sensors attached to the lower trunk and heel when subjects walked along a 15 m walkway at a self-selected preferred speed. The score was the sum of 10 gait parameter scores. The lower scores indicated more and worse abnormal gait patterns. In total, 24 older adults (10.8%) reported that they experienced pain after daily walking. According to the multiple logistic regression analyses, older adults with a lower total C-GAITS score had a significantly greater probability of having past experiences of pain after walking (odds ratio = 1.11, 95% confidence interval = 1.03–1.20). The findings of this study suggest that more and worse abnormal gait patterns among older adults in a clinical walking test are associated with an elevated degree of pain after daily walking.

## 1. Introduction

Walking is a major physical activity in daily life. Many people, especially older adults, experience musculoskeletal pain during walking [1]. Most causes of pain are osteoarthritis (OA) of the hip or knee or problems of the lower back or foot, which have a negative impact on gait [2,3,4,5]. There is robust evidence that pain interferes with a high-paced, regular, and smooth gait pattern [6,7,8,9]. For instance, a population-based study of community-dwelling older adults revealed that pain distribution was associated with slower gait speed and greater gait variability [6]. Our previous study revealed that moderate/severe pain in at least one site or pain of any intensity in multiple sites was associated with a lower regularity of trunk movement in community-dwelling older adults [7].

Abnormal gait patterns, such as lower-paced, variable, irregular, and uneven gait patterns, may have adverse effects on the body. Whether abnormal gait patterns cause pain has attracted little attention in the literature. To our knowledge, few studies have examined the relationship between gait patterns, including trunk movement patterns, and pain after walking the distance required to perform daily life activities among community-dwelling older adults. Trunk movement assessment is considered important because it locates the center of mass in the body and contributes to a great extent to efficient walking. A new method for gait assessment that sums up the scores derived from 10 gait parameters representing pace, variability, regularity, or smoothness of gait was recently reported [10]. The concept behind this method was that multiple abnormal gait patterns would overlap among older adults with severe gait problems. This newly developed method was highly sensitive to the decline in various functions among older adults, with the score representing this decline adequately [10]. We hypothesized that the score for the assessment of abnormal gait patterns during a clinical walking test would be associated with an elevated degree of pain after daily walking because multiple and worse abnormal gait patterns were believed to induce biased joint moments or inappropriate muscle activities during gait. Pain is one of the factors that have the highest impact on declining physical activity and the onset of disability in older adults [11,12]. Understanding the effect of gait patterns on pain in older adults would provide vital insights into the rehabilitation approach to improving pain.

The present study aimed to examine whether the score obtained after the assessment of abnormal gait patterns using inertial sensors during a clinical walking test was associated with experiencing an elevated level of pain after daily walking. A question-based assessment for pain was implemented as a preliminary study. 

## 2. Materials and Methods

### 2.1. Subjects

This study enrolled 234 subjects aged 65 years or older who lived independently in the community. They were recruited through community organizations for the elderly in two regions of the city of Kobe, Japan. The study sample size was set for practical reasons and a calculation for appropriate sample size was not performed. Exclusion criteria were cognitive impairment (Mini-Mental State Examination score < 20 [13] or Rapid Dementia Screening Test score < 7 [14]), having a disability that prevents walking without an assistive device, or failure to complete our assessment. After excluding 11 subjects, the data from 223 older adults were incorporated into the analyses. The background characteristics of the subjects were assessed using a self-reported questionnaire that included questions about age, gender, and comorbidities. 

### 2.2. Assessment of Pain

Pain experienced after daily walking was assessed by only asking a simple question about what the subject had experienced at that point, without applying an actual long walking test: “After you walked for 400 m or more, did you experience an elevated degree of pain compared with that at the beginning of walking?” Subjects answered using either YES or NO and were classified into two groups. We used the length of walking of 400 m as the distance required to perform daily life activities because this distance has been recognized as important for independent living and is often used as an index of mobility disability [15]. The musculoskeletal pain status was also assessed using other assessments.

### 2.3. Assessment of Gait Pattern

Gait pattern was assessed using the Comprehensive Gait Assessment using InerTial Sensor score (C-GAITS score), which was developed and validated by the authors [10]. This score was derived from the sum of 10 gait parameter scores calculated using data derived from the inertial sensors attached to the lower trunk and heel of the subjects during a clinical walking test.

Details of the methodology and calculation of the C-GAITS score are described elsewhere [10]. Briefly, the subjects walked along a smooth, horizontal walkway 15 m in length at a self-selected preferred speed. Inertial sensors (MVP-RF8; MicroStone, Nagano, Japan) were attached at the level of the L3 spinous process and heel of the subjects. Then, 10 gait parameter scores (walking speed, mean stride time, coefficient of variation (CV) of stride time, CV of swing time, autocorrelation coefficients (ACs) in three directions (vertical [VT], mediolateral [ML], and anteroposterior [AP]), and harmonic ratio (HR) in three directions) were calculated using the data captured by the inertial sensors. CV is calculated using the following formula and represents gait variability [16]:

CV = (standard deviation/mean) × 100 [%]

AC is an estimate of the regularity of a time series by cross-correlation with itself at a given time shift. AC is calculated using the formula given below; *i* indicates time, and *x*(*i*) indicates the acceleration data at time *i*.

$$\mathrm{AC}\left(m\right)=\frac{1}{N-\left|m\right|}\overset{N-\left|m\right|}{\underset{i=1}{\sum }}x\left(i\right)x\left(i+m\right)$$

It is independent of the amount of data managed and represents the regularity of trunk movement [17]. HR is calculated by digital Fourier transforms on the acceleration signals of each stride at the trunk. The HRs in the VT and AP directions are calculated as the sum of the amplitudes of the first 10 even harmonics divided by the sum of the amplitudes of the first 10 odd harmonics [18].

$$\mathrm{HR}\;\left(\mathrm{VT},\;\mathrm{AP}\right)=\frac{\sum even\;harmonics}{\sum odd\;harmonics}$$

In contrast, the HR in the ML direction is calculated as the sum of the amplitudes of the odd harmonics divided by the sum of the amplitudes of the even harmonics [18].

$$\mathrm{HR}\;\left(\mathrm{ML}\right)=\frac{\sum odd\;harmonics}{\sum even\;harmonics}$$

HR represents walking smoothness during gait and step-to-step asymmetry [18,19].

**Figure 1:**
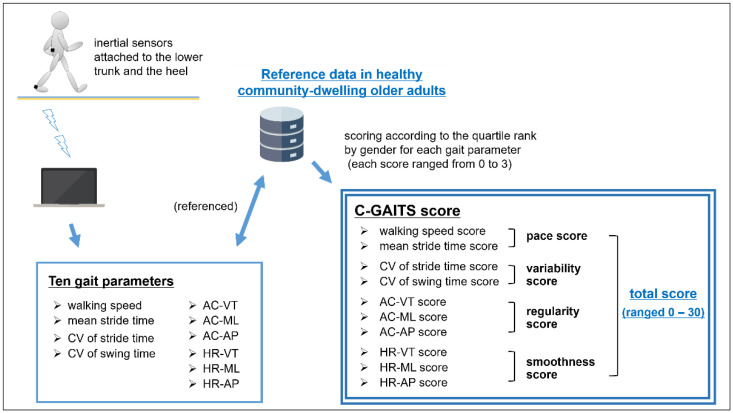
Overview of the Comprehensive Gait Assessment using the InerTial Sensor score (C-GAITS score).

For a uniform contribution of each gait parameter to the score, scoring was performed according to the quartile rank by gender for each gait parameter (i.e., scores ranged from 0 to 3) based on results in a large cohort of community-dwelling healthy older adults. A comprehensive total score was obtained by summing the various gait parameters (i.e., scores ranged from 0 to 30). The lowest scores indicated multiple abnormal gait patterns and poorer quality gait function. In addition, based on the results of the exploratory factor analysis, the score was composed of four subscores: pace score (the sum of scores for walking speed and mean stride time), variability score (the CV of stride time and swing time scores), regularity score (AC in the three direction scores), and smoothness score (HR in the three direction scores) (Figure 1). 

### 2.4. Statistical Analyses

Statistical analyses were performed using commercially available software (JMP13.2.0 J; SAS Institute Japan, Tokyo, Japan). Background characteristics and the C-GAITS score during a clinical walking test were compared based on the pain status after daily walking using an unpaired *t-*test or Fisher’s exact test. Multiple logistic regression analyses were then performed to adjust for age and gender. In the first model, pain after walking was a dependent variable, and the total C-GAITS score was an independent variable. In the second model, the subscores that were significantly related to pain after walking in the univariate analysis were included as independent variables. The overall level of statistical significance was set at 0.05.

## 3. Results

### 3.1. Characteristics of the Study Subjects

A total of 24 older adults (10.8%) reported that they experienced an elevated degree of pain after walking for 400 m or more. Participants reported pain in the knee (50.0%), lower back (25.0%), and lower limb (hip, toe, or thigh). The background characteristics of subjects in terms of pain after walking are presented in Table 1. 

### 3.2. Experiencing an Elevated Degree of Pain after Walking and C-GAITS Score

The results of the C-GAITS score by pain after daily walking are shown in Table 2. The subjects who experienced an elevated degree of pain after walking had a lower total C-GAITS score, pace subscore, and smoothness subscore than the subjects who did not experience an elevated degree of pain after walking.

In the multiple logistic regression analyses, older adults with a lower total C-GAITS score had a significantly greater probability of experiencing pain after walking (odds ratio (OR) = 1.11, 95% confidence interval (CI) = 1.03–1.20). The results of the second model showed that a lower pace score and a lower smoothness score were independently associated with a greater probability of experiencing pain after walking (OR = 1.34, 95% CI = 1.03–1.76; OR = 1.20, 95% CI = 1.00–1.45, resp.; c.f. Table 3).

## 4. Discussion

The present study demonstrated that, in community-dwelling older adults, a lower C-GAITS score, which was calculated from a clinical walking test, was associated with an assertive answer to the question of whether an elevated degree of pain was experienced after walking for 400 m or more. In addition, the two subscores of the C-GAITS score—the pace score and the smoothness score—were independently associated with a greater probability of pain following daily walking after adjustments for age and gender. Considering the evidence that pain affected gait patterns [6,7,8] and that the findings of this study demonstrated that more and worse abnormal gait patterns induce an elevated degree of pain after daily walking, we suggest that abnormal gait patterns and pain form a negative cycle.

These results are compatible with those of previous studies using a motion capture system. Boyer and Hafer reported that patients with knee OA who experienced an elevated degree of pain after 20 min of walking had greater joint moments and greater muscle co-activation at baseline than those who did not experience an elevated degree of pain and the control group [20]. Marriotto et al. showed that greater peak adduction moment and lower flexion and extension moments were independently associated with a greater likelihood of an increase in pain after a six-minute walk test [21]. Moreover, in patients with lumbar spinal stenosis, peak trunk tilt and pelvic tilt during walking could reportedly predict an aggravation of low back pain or leg pain after a six-minute walk test [22]. In patients with cerebral palsy, pelvic tilt during gait was found to be significantly associated with back pain, experienced mainly during prolonged walking [23]. Although these studies focused on patients with a specific disease, their results were comparable to ours in terms of demonstrating that gait patterns affected the elevated degree of pain after a relatively long walk.

The relationship between worse and more abnormal gait patterns during a walking test and pain after daily walking was partially explained in some previous studies that suggested that abnormal walking patterns exacerbated joint pain. In their 12-month prospective study, Tateuchi et al. reported that limited hip extension and limited external rotation angles during gait were associated with worsening hip pain in patients with hip OA [24]. In a prospective study of patients with knee OA, a varus thrust of the knee during gait, which is associated with a knee adduction moment, was related to worsening knee pain [25]. The subjects in our study included older adults with musculoskeletal problems and most of these subjects with pain after walking were already experiencing some degree of pain before walking. Thus, the results of this study expand the results of past studies [24,25]. Although we assessed spatiotemporal gait parameters and trunk movements that locate the center of mass, and neither joint moments nor muscle activations, the more and worse abnormal gait patterns were considered to affect biased joint loads or irregular muscle activations. These might cause an elevated degree of pain after walking; however, further studies are strongly warranted.

The pace score and the smoothness score were independently associated with a greater probability of pain after daily walking. The pace score includes walking speed and mean stride time, both of which represent overall gait performance. The smoothness score includes HRs of trunk acceleration in the three directions. The HR represents the smoothness of movements “within stride” and correlates with step-to-step symmetry [18,19]. Gait consists of repeated movement; thus, uneven and asymmetrical gait patterns induce biased mechanical stress on some parts of the body, which may result in pain after daily walking.

This preliminary study has some limitations. First, the primary outcome, pain after daily walking, was assessed by just asking a simple question. To confirm the association, assessments that include real walking experiments (for instance, the six-minute walk test) should be implemented. However, we asked the subjects about an “elevated degree of pain compared with that at the beginning of walking”, and the association between gait pattern and pain was ascertained regardless of whether they had any musculoskeletal pain before walking. Thus, it is possible that worse and more abnormal gait patterns exacerbate pain after walking. Secondly, the number of subjects was not large enough to enable multivariate statistical analyses that consider other confounding factors. Thirdly, a few subjects who used assistive devices were excluded from our analysis to eliminate the effect of using assistive devices on gait data. Although the effect of excluding these subjects was minimal because of a small number of subjects, the people with pain that could limit functions were the very subjects to study. To extrapolate and generalize our results, further studies considering other confounding factors and including subjects who have worse gait performance are required.

## 5. Conclusions

The present study demonstrated the association between lower C-GAITS scores and an assertive answer to the question of whether an elevated degree of pain was experienced after walking for 400 m or more among community-dwelling older adults. The results suggested that more and worse abnormal gait patterns in a clinical walking test were associated with an elevated degree of pain after daily walking. Considering past evidence, abnormal gait pattern and pain may form a negative cycle in older adults, abnormal gait patterns is an important pain-inducing factor, and pain affects gait pattern.

## Figures and Tables

**Table 1:** Characteristics of subjects based on the experience of an elevated degree of pain after daily walking.

Variable		Did Not Experience an Elevated Degree of Pain after Daily Walking (*n* = 199, 89.2%)	Experienced An Elevated Degree of Pain after Daily Walking (*n* = 24, 10.8%)	*p*-Value
Age	(y)	74.6 ± 5.4	75.8 ± 6.6	0.35
Female	(*n* (%))	115 (57.8)	18 (75.0)	0.13
Body mass index	(kg/m^2^)	23.1 ± 2.8	23.8 ± 2.9	0.27
Medical history	(*n* (%))			
Hypertension ^1^		79 (39.9)	13 (54.2)	0.19
Diabetes ^1^		21 (10.6)	5 (20.8)	0.17
Arteriosclerosis ^2^		1 (0.5)	0 (0.0)	1.00
Stroke ^1^		5 (2.5)	2 (8.3)	0.17
Hip OA ^1^		5 (2.5)	5 (20.8)	0.002
Knee OA ^1^		26 (13.1)	8 (33.3)	0.016
Spinal canal stenosis ^1^		18 (9.1)	3 (12.5)	0.48
Other spinal disease ^2^		18 (9.1)	5 (20.8)	0.085
Any musculoskeletal pain ^1^	(*n* (%))	104 (52.5)	21 (87.5)	<0.001
Lower extremity pain ^1^	(*n* (%))	59 (29.8)	16 (66.7)	<0.001
Low back pain ^1^	(*n* (%))	40 (20.2)	13 (54.2)	<0.001

Values are means ± standard deviation or percentages. *p*-values were calculated using an unpaired *t*-test or Fisher’s exact test by answering a question about whether the participant experienced an elevated degree of pain after walking. ^1^ Total number of data points is 222 of 223 because of missing data. ^2^ Total number of data points is 221 of 223 because of some missing data. OA: osteoarthritis.

**Table 2:** C-GAITS score of subjects based on the experience of an elevated degree of pain after walking.

Variable	Did Not Experience an Elevated Degree of Pain after Daily Walking (*n* = 199, 89.2%)	Experienced An Elevated Degree of Pain after Daily Walking (*n* = 24, 10.8%)	*p*-Value
Total score	14.4 ± 6.3	10.3 ± 6.1	0.003
Subscores			
Pace score	2.8 ± 2.0	1.5 ± 1.5	0.004
Variability score	3.2 ± 1.9	2.6 ± 2.3	0.17
Regularity score	3.6 ± 2.8	2.9 ± 2.9	0.21
Smoothness score	4.8 ± 2.6	3.3 ± 2.5	0.007

Values are means ± standard deviation or percentages. *p*-values were calculated using an unpaired *t*-test by answering a question about whether the participant experienced an elevated degree of pain after daily walking. C-GAITS score: Comprehensive Gait Assessment using InerTial Sensor score.

**Table 3:** Odds ratio for elevated degree of pain after daily walking in multiple logistic regression analyses.

Independent Variable	Model 1			Model 2		
	Odds Ratio	95% CI	*p*-Value	Odds Ratio	95% CI	*p*-Value
C-GAITS score						
Total score	0.90	0.83–0.97	0.008			
Pace score				0.74	0.57–0.97	0.032
Smoothness score				0.83	0.69–0.99	0.045
Age	1.02	0.98–1.11	0.68	1.01	0.93–1.10	0.76
Sex (female)	2.12	0.79–5.68	0.12	1.70	0.62–4.64	0.30

CI: confidence interval; C-GAITS score: Comprehensive Gait Assessment using InerTial Sensor score.

## Data Availability

The data presented in this study are only available to members of this project. The data will not be shared with others because consent for data sharing was not obtained from the participants.
